# Abnormal Mitochondrial cAMP/PKA Signaling Is Involved in Sepsis-Induced Mitochondrial and Myocardial Dysfunction

**DOI:** 10.3390/ijms17122075

**Published:** 2016-12-10

**Authors:** Remi Neviere, Florian Delguste, Arthur Durand, Jocelyn Inamo, Eric Boulanger, Sebastien Preau

**Affiliations:** 1 Département de Physiologie, Faculté de Médecine, Université Lille, 1 Place de Verdun, F-59000 Lille CEDEX 59045, France; 2 INSERM LIRIC U995/Team “Glycation: From Inflammation to Aging”, Université Lille, F-59000 Lille, France; florian.delguste@univ-lille2.fr (F.D.); durand.arthur@gmail.com (A.D.); eric.boulanger@univ-lille2.fr (E.B.); seb.preau@gmail.com (S.P.); 3 Pôle Réanimation Médicale, CHU Lille, Bd Pr Leclercq, F-59000 Lille, France; 4 Département de Cardiologie, CHU Martinique, Faculté de Médecine, Université des Antilles, F-97200 Fort de France, France; Jocelyn.Inamo@chu-fortdefrance.fr

**Keywords:** mitochondria respiration, cyclic adenosine monophosphate (cAMP), protein kinase A, soluble adenylyl cyclase, phosphodiesterase, isolated heart, sepsis

## Abstract

Adrenergic receptors couple to Gs-proteins leading to transmembrane adenylyl cyclase activation and cytosolic cyclic adenosine monophosphate (cAMP) production. Cyclic AMP is also produced in the mitochondrial matrix, where it regulates respiration through protein kinase A (PKA)-dependent phosphorylation of respiratory chain complexes. We hypothesized that a blunted mitochondrial cAMP-PKA pathway would participate in sepsis-induced heart dysfunction. Adult male mice were subjected to intra-abdominal sepsis. Mitochondrial respiration of cardiac fibers and myocardial contractile performance were evaluated in response to 8Br-cAMP, PKA inhibition (H89), soluble adenylyl cyclase inhibition (KH7), and phosphodiesterase inhibition (IBMX; BAY60-7550). Adenosine diphosphate (ADP)-stimulated respiratory rates of cardiac fibers were reduced in septic mice. Compared with controls, stimulatory effects of 8Br-cAMP on respiration rates were enhanced in septic fibers, whereas inhibitory effects of H89 were reduced. Ser-58 phosphorylation of cytochrome c oxidase subunit IV-1 was reduced in septic hearts. In vitro, incubation of septic cardiac fibers with BAY60-7550 increased respiratory control ratio and improved cardiac MVO_2_ efficiency in isolated septic heart. In vivo, BAY60-7550 pre-treatment of septic mice have limited impact on myocardial function. Mitochondrial cAMP-PKA signaling is impaired in the septic myocardium. PDE2 phosphodiesterase inhibition by BAY60-7550 improves mitochondrial respiration and cardiac MVO_2_ efficiency in septic mice.

## 1. Introduction

Activation of G protein–coupled receptors represents the primary mechanism to increase cardiac function under stress [1]. Following binding of agonist ligands, coupling of β-adrenergic receptors to stimulatory Gs proteins induces transmembrane adenylyl cyclase (tmAC) activation, which catalyzes conversion of adenosine triphosphate (ATP) to 3′,5′-cyclic adenosine monophosphate (cAMP) [1,2]. Binding of cAMP on regulatory subunits of the inactive tetrameric protein kinase A (PKA) causes regulatory subunits to detach, exposing the two catalytic subunits [3]. Free catalytic subunits can then catalyze the transfer of ATP terminal phosphates at serine or threonine residues of many proteins involved in heart contractility and remodeling [4]. The key feature of cAMP-PKA transduction system is compartmentalization of its signaling via physical interactions with A-kinase anchoring proteins and cAMP degrading phospho-diesterases (PDE), controlling cAMP levels within subcellular microdomains [3,4]. Spatiotemporal compartmentalization of cAMP signaling elicits phosphorylation of proteins involved in contraction, including Na^+^/Ca^2+^ exchanger and l-type Ca^2+^ channel at the sarcolemma [5], and phospholamban and ryanodine receptors at the sarcoplasmic reticulum [6,7].

Although kinetic of catecholamine release and adrenoceptor signaling may be quite different in human sepsis, small rodent models of polymicrobial sepsis typically induce excessive adrenergic stimulation, rapid downregulation of β-adrenergic receptors and abnormal post-receptor cAMP-PKA signaling [8,9,10]. Uncoupling of β-adrenergic receptors may further impair post receptor transduction pathway via stimulatory Gs and inhibitory Gi protein modifications [8,9,10]. Abnormal β-adrenergic receptor signaling is responsible for cardiac depression through aberrant cytosolic calcium fluxes and impaired electromechanical coupling at the myofibrillar level [8,9,10]. As a different concept, we and other groups have proposed that the septic cardiac contractile dysfunction may be related, at least in part, to mitochondrial and bioenergetic dysfunction [11,12,13]. Mitochondrial metabolism can be affected by sepsis in various ways, which include diminished activities of Krebs cycle enzymes, reduced electron transfer chain oxidative capacity, mitochondrial proton leak and mitochondrial uncoupling [11]. Here, we propose that abnormal cAMP-PKA signaling would also participate in sepsis-induced mitochondrial dysfunction.

**Figure 1:**
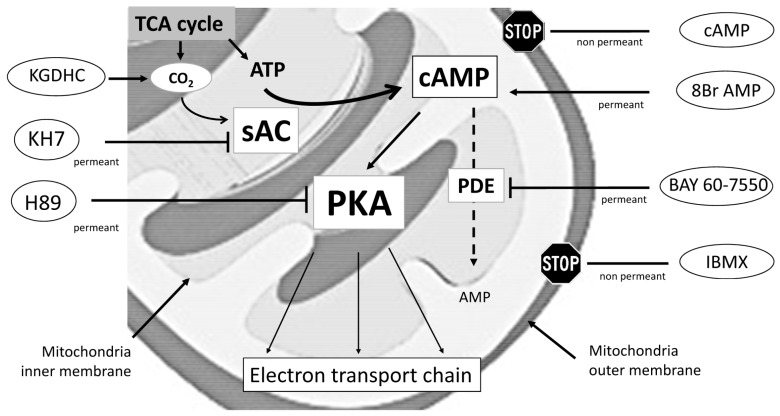
Schematic description of electron transfer chain control by mitochondrial cAMP-PKA signaling. TCA: acid tricarboxylic acid (TCA) cycle or the Krebs cycle; sAC: soluble adenylyl cyclase; PKA: protein kinase A; PDE: phosphodiesterases; cAMP: cyclic adenosine monophosphate. Solid arrow indicates “stimulatory effects”; T-bar indicates “inhibitory effects”; dotted arrow indicates “decomposition”.

The direct role of cAMP-PKA signaling in regulation of mitochondrial oxidative phosphorylation has recently begun to emerge [5,14,15,16]. Central to this new paradigm is that cytosolic cAMP does not cross over the inner mitochondrial membrane. Instead, elevation of mitochondrial cAMP is attributed to a unique form of mammalian soluble adenylyl cyclase (sAC) structurally, molecularly, and biochemically distinct from transmembrane adenylyl cyclase (tmAC). The sAC localized within the mitochondrial matrix is directly stimulated by CO_2_/HCO_3_^−^, but not by forskolin that only stimulates tmAC [14,16,17]. Numerous reports revealed that PKA localized at an intermembrane space and matrix was the effector of cAMP in mitochondria, which modulates respiration via phosphorylation of electron transfer chain complexes [18]. Serine/threonine PKA-dependent phosphorylation of complex-I and cytochrome c oxidase (CcOX) subunit IV-1 can increase their activities [19], whereas phosphorylation of CcOX catalytic subunit I on tyrosine residue via a PKA-regulated tyrosine kinase inhibits oxidative phosphorylation [20,21]. Overall, cAMP is generated by sAC in the mitochondrial matrix, where it balances mitochondrial metabolism following PKA-dependent phosphorylation of electron transfer chain complexes, coupling CO_2_ generation in the Krebs cycle with activity of oxidative phosphorylation machinery (Figure 1) [15].

Whether sepsis would alter mitochondrial cAMP-PKA signaling is unknown. Our objective was to test whether sepsis induced by fecal peritonitis in mice would alter mitochondrial cAMP-PKA signaling and PKA-dependent control of oxidative phosphorylation. We tested whether agents altering the cAMP-PKA signaling pathway would help regulate mitochondrial respiration in cardiac fibers of sham and septic mice. Next, we tested whether PDE inhibitors would increase cardiac cAMP levels and cardiac efficiency in isolated hearts of septic mice. Eventually, the effects of PDE2 inhibitor on heart contractile function were evaluated in vivo in septic mice.

## 2. Results

### 2.1. Locally Produced cAMP Stimulates Mitochondria Respiration and Increases Ser-58 CcOX Phosphorylation in Control Cardiac Fibers

**Figure 2:**
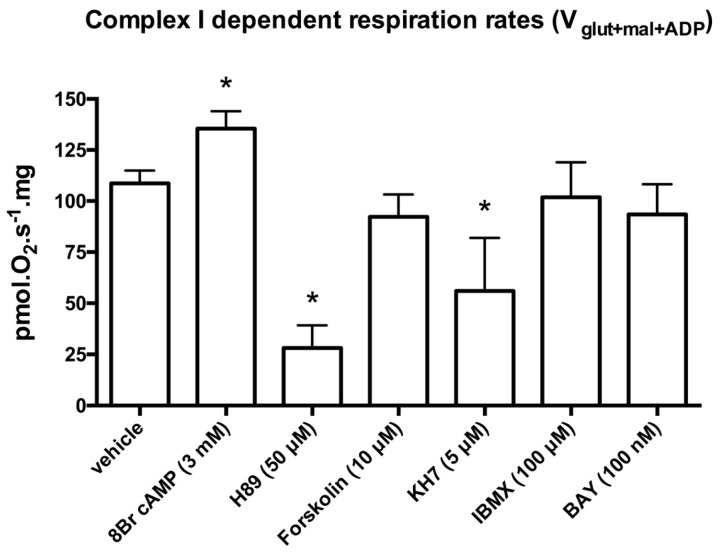
Mitochondrial complex I dependent “state 3” (V_glut+mal+ADP_) respiration rates (pmol·O_2_·s^−1^·mg) of permeabilized cardiac fibers in control (vehicle) and after incubation for 30 min with 8Br cAMP; H89; KH7; 3,7-dihydro-1-methyl-3-(2-methylpropyl)-1*H*-purine-2,6-dione (IBMX); or BAY 60-7550 are displayed. See Materials and Methods for details. Data are mean ± standard deviation (SD). Results were analyzed with one-way ANOVA and Bonferroni’s multiple comparison post hoc adjustment (*n* = 8 in each group; * indicates adjusted *p* < 0.007 versus vehicle).

**Figure 3:**
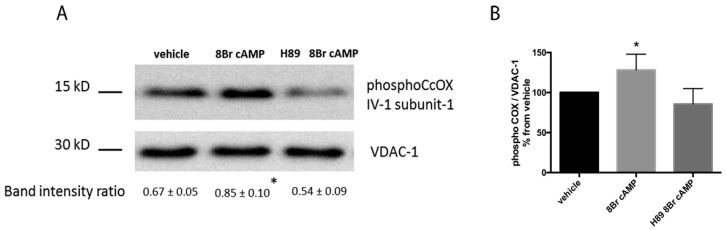
Representative Western blot images showing mitochondrial protein levels of Ser-58 phosphorylated cytochrome c oxidase (CcOX) subunit IV-1 of control mitochondrial fractions after incubation with 8Br cAMP with or without H89 pre-exposure. Results of densitometric quantification of blots normalized for voltage-dependent anion channel (VDAC)-1, (band intensity ratio) are displayed as mean ± SD (**A**); and expressed as percent changes (**B**). Results were analyzed with one-way ANOVA and Bonferroni’s multiple comparison post hoc adjustment (*n* = 6; * indicates adjusted *p* < 0.01 versus vehicle).

We measured oxygen consumption as an indicator of mitochondrial respiratory chain function in permeabilized cardiac fibers. In control cardiac fiber preparations, substrates and inhibitors used to alter mitochondria cAMP-PKA signaling had no effects on V_glut+mal_ respiratory rates (data not shown). 8Br-cAMP (3 mM), a cAMP membrane-permeant analog, resulted in a 25% ± 7% increase of V_glut+mal+ADP_ respiration rates (Figure 2). Conversely, the selective PKA catalytic subunit inhibitor H89 (50 μM) resulted in a 73% ± 9% decrease of V_glut+mal+ADP_ respiration rates (Figure 2). Activation of trans-membrane isoforms of AC by forskolin had non-significant effects on V_glut+mal+ADP_ respiration rates (Figure 2). Activation of sAC, which is not regulated by heterotrimeric G proteins or forskolin, can be activated by CO_2_. The CO_2_-generating system α-ketoglutarate dehydrogenase complex (KGDHC) increased *N*,*N*,*N*′,*N*′-tetramethyl-*p*-phenylenediamine dihydrochloride (TMPD)/ascorbate-dependent respiration rates by 34% ± 10% (60 ± 5 vs. 80 ± 6 pmol·O_2_·s^−1^·mg; *p* < 0.001), suggesting that sAC activation stimulated cytochrome c oxidase (CcOX)-dependent respiration. Conversely, KH7 (5 μM), a sAC inhibitor, reduced V_glut+mal+ADP_ respiration rates by 47% ± 14% (Figure 2). Non permeant PDE inhibitor 3,7-dihydro-1-methyl-3-(2-methylpropyl)-1*H*-purine-2,6-dione (IBMX) (100 μM) and BAY 60-7550 (100 nM) had no effects on V_glut+mal+ADP_ respiration rates (Figure 2), suggesting that PDE inhibition was insufficient to alter mitochondrial cAMP concentration in controlling cardiac fibers. Incubation of mitochondrial fractions with 8Br-cAMP increased Ser-58 phosphorylated CcOX (Figure 3). Pre-incubation of mitochondrial fractions with the PKA inhibitor H89 prevented 8Br-cAMP-induced Ser-58 CcOX phosphorylation (Figure 3), suggesting that CcOX phosphorylation was a PKA-dependent event.

### 2.2. Blunted Mitochondrial cAMP-PKA Signaling in the Septic Heart Is Improved by PDE2 Inhibition

Cecal ligation puncture (CLP)-operated mice developed polymicrobial sepsis with no mortality at 24 h. A Murine Sepsis Score (MSS) was used to assess disease severity in sham and CLP–operated mice and was calculated at 4 h and 24 h. In sham mice, MSS was 1 (1–2) and 1 (1–1) (median (25%–75% percentile)) at 4 h and 24 h, respectively. In CLP mice, MSS was 12 (10.5–15.0) and 17 (15.25–19.50) (median (25%–75% percentile)) at 4 h and 24 h, respectively.

**Figure 4:**
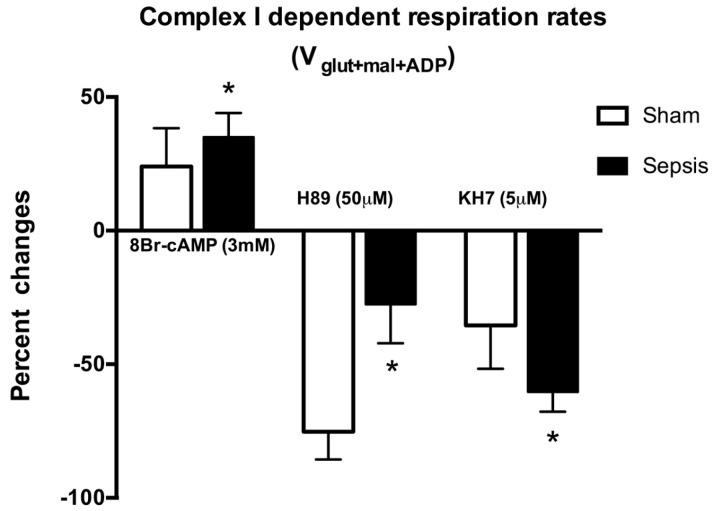
Changes of mitochondrial complex I-dependent “state 3” (V_glut+mal+ADP_) respiration rates in sham and sepsis (cecal ligation and puncture (CLP)) in permeabilized cardiac fibers in response to 8Br-cAMP; H89; or KH7. See Materials and Methods for details. Data are expressed as percent of change and displayed as mean ± SD. Results were analyzed with one-way ANOVA and Bonferroni’s multiple comparison post hoc adjustment (*n* = 8 in each group; * indicates adjusted *p* < 0.01 versus sham).

**Figure 5:**
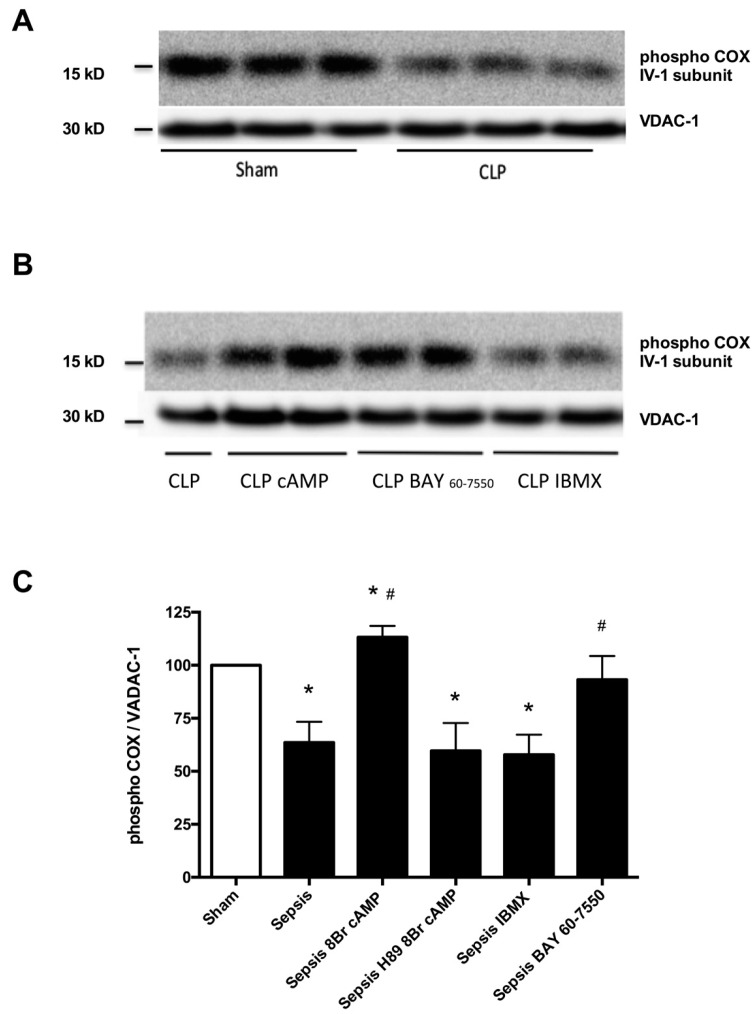
Representative Western blot images showing protein levels of Ser-58 phosphorylated cytochrome c oxidase (CcOX) subunit IV-1 of mitochondrial fractions isolated from three independent experiments in sham and sepsis (CLP) (**A**); representative Western blot images showing protein levels of Ser-58 phosphorylated cytochrome c oxidase (CcOX) subunit IV-1 (**B**); and densitometric quantification (**C**) of protein levels of Ser-58 phosphorylated cytochrome c oxidase (CcOX) subunit IV-1 normalized for VDAC-1 of mitochondrial fractions isolated from sepsis (CLP) mice after incubation with 8Br-cAMP alone and with H89 pre-exposure and after incubation IBMX; or BAY 60-7550. Results are expressed as percent changes from sham levels and displayed as mean ± SD. And analyzed with one-way ANOVA and Bonferroni’s multiple comparison post hoc adjustment (*n* = 6 in each group; * indicates *p* < 0.05 versus sham; ^#^ indicates adjusted *p* < 0.008 versus Sepsis).

Compared with control, V_glut+mal+ADP_ respiration rates of permeabilized cardiac fibers were reduced in the septic mice (103.6 ± 13.4 vs. 87.7 ± 4.5 pmol·O_2_·s^−1^·mg; *p* = 0.012) (Table 1). Compared with controls, magnitude of 8Br-cAMP (3 mM) stimulatory effects on mitochondrial respiration was higher in septic cardiac fibers (24% ± 10% vs. 36% ± 7%; *p* = 0.01) (Figure 4). Conversely, inhibitory effects of H89 (50 μM) on mitochondrial respiration were less important in septic cardiac fibers than in controls (27% ± 12% vs. 75% ± 7%; *p* = 0.001) (Figure 4). Compared with controls, soluble AC inhibitor KH7 (5 μM) resulted in larger mitochondrial respiration decreases in septic cardiac fibers (60% ± 6% vs. 35% ± 14%; *p* = 0.0003) (Figure 4). IBMX (100 μM) had no effects on V_glut+mal_ and V_glut+mal+ADP_ respiration rates in septic cardiac fibers (Table 1). BAY 60-7550 (100 nM) had no effects on V_glut+mal+ADP_ respiration rates but decreased V_glut+mal_ respiration rates, thus improving respiratory control ratio in septic cardiac fibers (Table 1). Consistent with blunted cAMP-PKA signaling, we found that phosphorylation of Ser-58 of CcOX subunit IV-1 protein was reduced in septic hearts compared with sham hearts (Figure 5A). Incubation of mitochondrial fractions with 8Br-cAMP increased Ser-58 phosphorylated CcOX, which was prevented by H89 pre-incubation (Figure 5B). BAY 60-7550 partially prevented CcOX subunit IV-1 protein dephosphorylation in septic permeabilized cardiac fiber, whereas IBMX had no effects (Figure 5B).

**Figure 6:**
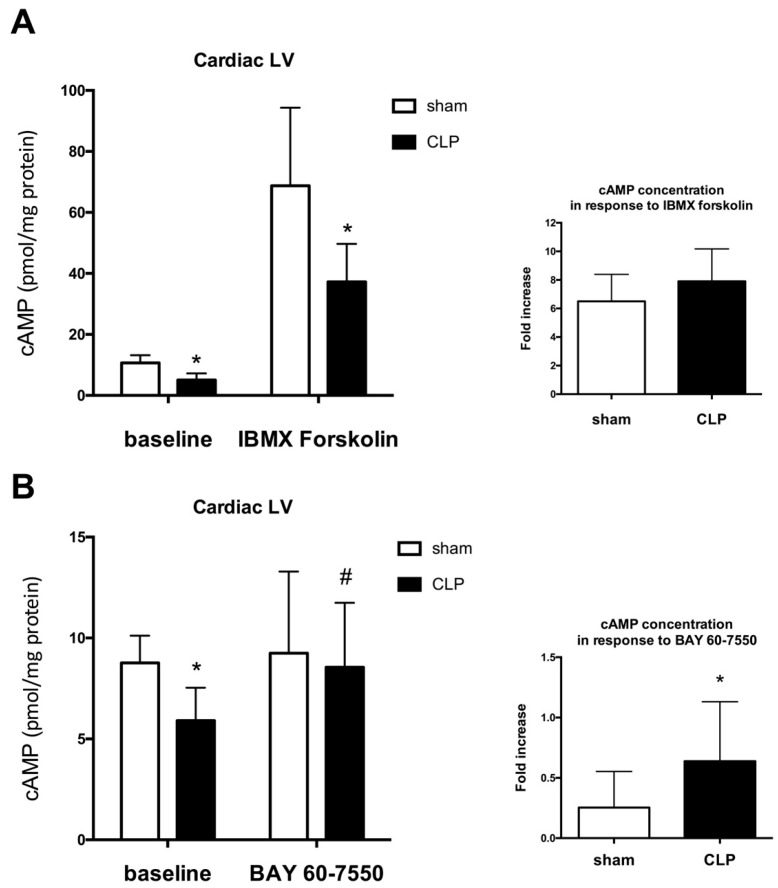
Effects of phophosdiesterase (PDE) inhibitors; IBMX (**A**); or BAY 60-7550 (**B**) on **left** ventricle (LV) cAMP concentrations (pmol/mg protein) in control and CLP sepsis. See Materials and Methods for details. Data (mean ± SD) are expressed in pmol/mg protein (**left** panels) and percent of changes from baseline conditions (**right** panels). Results were analyzed with one-way ANOVA and Bonferroni’s multiple comparison post hoc adjustment (*n* = 8 per group; * indicates *p* < 0.05 versus control group; ^#^ indicates *p* < 0.05 versus control baseline).

**Figure 7:**
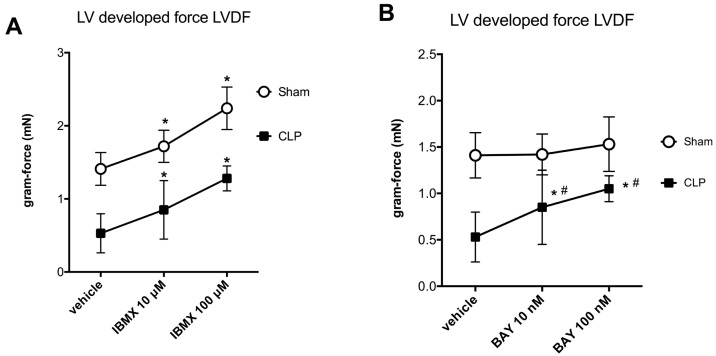
Effects of perfusion of phophosdiesterase (PDE) inhibitors; IBMX (**A**); or BAY 60-7550 (**B**) on **left** ventricle developed force (LVDF; gram.force in mN) in isolated hearts of control and CLP sepsis. See Materials and Methods for details. Data are mean ± SD. Results were analyzed with two-way ANOVA (dose response—group mice) and Bonferroni’s multiple comparison post hoc adjustment (*n* = 8 per group; * indicates *p* < 0.05 versus vehicle. ^#^ indicates adjusted *p* < 0.01 versus control group).

In isolated septic hearts, myocardial efficiency (left ventricular developed force (LVDF) × heart rate/MVO_2_) was markedly reduced as a result of larger decreases of LVDF compared with decreases in myocardial oxygen consumption MVO_2_ (Table 2). Basal heart cAMP levels were decreased in septic hearts compared with sham hearts. IBMX (100 μM), a non-selective PDE inhibitor, induced similar fold increases in cAMP levels in sham and septic hearts (Figure 6A). IBMX coronary perfusion dose-dependently increased LVDF in sham and septic hearts (Figure 7A). Expressed as percent of increase, dose-response effects of IBMX perfusion on heart contractile function were similar in septic hearts compared to sham hearts (Figure 7A). IBMX perfusion had no effects on sepsis-induced cardiac inefficiency as MVO_2_ increased proportionally with LVDF (Table 2). Heart perfusion with 100 nM BAY 60-7550 had no effects on basal heart cAMP levels in sham hearts, but significantly increased heart cAMP levels in septic hearts (Figure 6B). BAY 60-7550 (100 nM) had no inotropic effects in sham hearts, but dose-dependently increased LVDF in septic hearts (Figure 7B). Heart perfusion with 100 nM BAY 60-7550 improved cardiac efficiency in septic hearts (Table 2).

In vivo experiments were performed in mice to evaluate effects of PDE2 inhibition. BAY 60-7550 administered either as pre-treatment or treatment had no effects on myocardial function in sham mice (Table 2). BAY 60-7550 (0.5, 1 and 5 mg/kg) administered by intraperitoneal (i.p.) injection immediately before surgery had no effects in septic mice (data not shown). Pre-treatment with BAY 60-7550 (1 mg/kg i.p. for three consecutive days prior surgery) significantly reduced coronary perfusion pressure and improved cardiac efficiency (Table 2).

## 3. Discussion

New findings of this study are twofold. First, our results suggest that oxidative phosphorylation defects induced by sepsis may be attributed to abnormal mitochondrial cAMP-PKA signaling and reduced Ser-58 phosphorylation of cytochrome c oxidase (CcOX) subunit IV-1. Second, inhibition of PDE2A, a cAMP degrading phosphodiesterase localized within the mitochondrial matrix, improved respiratory control ratio of cardiac fibers and myocardial efficiency in septic hearts. Of note, in vivo treatment by PDE2 inhibitor had no effects in septic cardiomyopathy, which may question the restorative role of BAY 60-7550 in models of aggressive multi bacterial sepsis.

We took advantage of permeabilized cardiac fiber technique, in which cytosol is lost and mitochondria structures remain intact, allowing for rapid equilibration with the incubation solutions [22]. Our results confirm previous studies [5,14,15,16] showing that PKA localized at an intermembrane space and matrix is the effector of cAMP, which modulates mitochondrial respiration via phosphorylation of electron transfer chain complexes (Figure 1A). cAMP is produced within mitochondria by a HCO_3_/CO_2_ responsive soluble adenylyl cyclase, which couples CO_2_ generation in the Krebs cycle with oxidative phosphorylation machinery activity [14,15]. In line, we first found that inhibition of sAC decreased ADP-coupled mitochondrial respiration, whereas the CO_2_-generating α-ketoglutarate dehydrogenase system stimulated CcOX-dependent respiration rates. Second, we found that elevation of mitochondrial 8Br-cAMP levels increased ADP-coupled mitochondrial respiration, whereas PKA inhibition by H89 had the opposite effect. Interestingly, increases of Ser-58 CcOX subunit IV-1 phosphorylation induced by 8Br-cAMP were prevented by H89, which confirms that CcOX was phosphorylated in a PKA-dependent manner [19]. Before one concludes that cAMP levels can regulate mitochondria function, it is important to consider mechanisms by which cAMP signaling is switch off. Previous studies have consistently identified PDE2A activities in the mitochondrial matrix, inhibition of which stimulates oxidative phosphorylation [16,23,24]. In our study, PDE2A inhibition by BAY 60-7550 had no effects on mitochondrial respiration, suggesting that inhibition of cAMP degradation failed to sufficiently increase cAMP in control conditions. These unexpected results may be related to the complex regulation of CcOX, which is accomplished in various ways including expression of tissue-specific isoforms, reversible phosphorylation subunits and interactions with small molecules such as thyroid hormones and ATP. So-called allosteric ATP-inhibition of CcOX at high ATP/ADP ratio is only active when CcOX is phosphorylated. Of note, reversible PKA-mediated phosphorylation of Ser-58 of CcOX subunit IV-1 was recently found to prevent allosteric ATP-inhibition, as Ser-58 is part of the allosteric binding site for ATP on the matrix side of CcOX subunit IV-1. Hence, depending on baseline levels of ATP and CcOX phosphorylation, low cAMP increases by PDE2A inhibition may be insufficient to alter CcOX activity [23,24].

In sepsis, excessive increase of circulating catecholamines induces downregulation of β-adrenergic receptors and impaired post-receptor cAMP-PKA signaling in cardiomyocytes [25]. In addition, cardiac expression of stimulatory Gs-proteins is decreased in sepsis resulting in decreased activity of tmAC and reduced levels of cAMP [26,27,28]. Myocardial mRNA transcriptomic study recently confirmed that sepsis is accompanied with reduced transcript abundance of β-adrenergic receptors, tmAC isoforms and PKA subunits [29]. Whether cAMP-PKA signaling is impaired within mitochondria of septic heart is unknown. In the present study, blunted cAMP-PKA pathway was suggested indirectly by functional observations. In septic cardiac fibers, PKA inhibitory effects of H89 on mitochondrial respiration were reduced, whereas magnitude of mitochondrial respiration increases in response to cAMP replenishment were increased. These results were interpreted as reduced cAMP-PKA activation because H89 can only inhibit activities of catalytic subunits previously released from PKA holoenzyme [30]. Impaired PKA catalytic subunit activity was further suggested by reduced Ser-58 phosphorylation of CcOX subunit IV-1 in septic mice. Previous studies have shown that of CcOX phosphorylation at different sites may have the opposite effect on mitochondrial function (20,21,31). For example, Tyr-304 phosphorylation of CcOX subunit-I may strongly inhibit mitochondria respiration in acute inflammation [20,21]. In contrast, inhibition of PDE increases CcOX activities in septic mice, suggesting that cAMP/PKA-dependent CcOX phosphorylation may improve mitochondria respiration [31].

PDE inhibition has the potential to attenuate organ dysfunction induced by lipopolysaccharide (LPS) challenge and sepsis through increased intracellular cAMP levels [32,33,34]. PDE inhibitors reduce levels of pro-inflammatory and pro-oxidant mediators released by LPS-stimulated immune competent cells [32]. PDE inhibition reduced systemic inflammation, microvascular barrier dysfunction and organ injury in experimental sepsis [33,34]. Our study revealed that IBMX, a nonspecific inhibitor of PDE, and BAY 60-7550, a specific PDE2 inhibitor, had contrasting effects on myocardial function in septic mice. IBMX had no effects on impaired mitochondrial respiration, CcOX phosphorylation and myocardial dysfunction in septic cardiac fibers. In contrast, BAY 60-7550 reduced uncoupled (V_glut+mal_) respiration rates, while coupled respiratory rates were unchanged leading to higher respiratory control ratio. BAY 60-7550 increased CcOX phosphorylation in mitochondrial fractions of septic cardiac fibers and improved cardiac MVO_2_ efficiency in isolated septic hearts. These findings are consistent with previous studies supporting that PDE inhibition reduces mitochondrial reactive oxygen species production, inner membrane leak, and swelling, thus preventing mitochondrial uncoupling in oxidative stress conditions [35]. To ascertain that PDE2A inhibition may target mitochondrial function in vivo, we tested whether BAY 60-7550 would improve heart function in septic mice. Pre-treatment of septic mice by BAY 60-7550 had no effects on LV contractile force, but significantly reduced coronary perfusion pressure and improved cardiac efficiency. Absence of effects of PDE2 inhibition in vivo may be related to the known effects of BAY 60-7550 on nitric oxide bioavailability. For example, in LPS-treated macrophages, PDE2 inhibition has been associated with enhanced nitric oxide via increased inducible nitric oxide expression, which may lead coronary vasodilation and alter mitochondrial function [36].

### Study Limitation

Although a functional cAMP-PKA pathway in cardiac mitochondrial was identified in septic mice, our study has several limitations. First, novelty and clinical relevance of our study is clearly limited by the absence of major effects of PDE2 inhibition in vivo. In our study, only pre-treatment with the PDE inhibitor BAY 60-7550 for three consecutive days prior to surgery showed some effects on cardiac efficiency, but no effects could be shown by a co-treatment approach. Of note, PDE2 inhibition in vivo had no effects on heart contractile dysfunction in CLP-mice. These results are in contrast with several studies reporting attenuation of organ dysfunction in sepsis by PDE inhibition. Second, PDE2 inhibition using BAY 60-7550 is not strictly mitochondria-specific, so that conclusions from the ex vivo or in vivo studies cannot be fully justified, since the observed effects can be attributed to a broad range of cellular PDE2 effects. Moreover, different types of PDE (PDE2, PDE3 and PDE4) are present in the mitochondria, inhibition of which may not be achieved by BAY 60-7550, a specific PDE2 inhibitor. Hemodynamic effects of PDE2 inhibition in vivo were not as strong as it was in ex vivo preparations. Mitigated effects of BAY 60-7550 in septic mice may be related to the confounding effects of hypothermia induced by CLP, which may profoundly alter the cardiovascular response to adrenergic stimulation [37].

## 4. Materials and Methods

### 4.1. Animal Care and Model of Sepsis

Swiss-type mice were purchased from Charles River Laboratories (Lille, France). Mice were housed on a 12-h light/dark cycle with ad libitum access to water and food. Male mice were studied at the age of 10 to 12 weeks. Investigations were conducted with the approval of our Institute review board (C2EA-75, 15 January 2015 Ethical Committee in Animal Experimentation of Nord-Pas-de-Calais, France) and conformed to the EU directive 2010/63/EU of the European parliament and the Animal Research: Reporting of In Vivo Experiments *ARRIVE* guidelines developed by the National Center for the Replacement, Refinement, and Reduction of Animals in Research (nc3rs). CLP was performed to induce intra-abdominal peritonitis as a relevant model of sepsis. Mice were slowly induced to anesthesia with 2% isoflurane and maintained at 1% isoflurane during surgical procedure. Mice were shaved and scrubbed with betadine. A middle abdominal incision was made and the cecum was mobilized. The cecum was ligated at its base below the ileocecal valve using 4-0 silk sutures and punctured twice with a 21-gauge needle. The cecum was gently squeezed to expel some fecal material and returned to the peritoneal cavity. Abdominal musculature and skin were closed in two layers using silk sutures. Mice were resuscitated by 1.0-mL subcutaneous injection of 0.9% saline. Sham-operated animals were handled identically, without ligation and puncture. Murine sepsis score was routinely calculated to monitor sham-operated and septic animals [38]. Postoperative analgesia was performed by buprenorphin (0.05 mg/kg) administered by subcutaneous (s.c.) injection. Where indicated, in vivo treatment was performed in sham and CLP mice by a single intraperitoneal injection of BAY 60-7550 immediately after surgery. In vivo pretreatment was performed in sham and CLP mice by injection of BAY 60-7550 once a day for 3 consecutive days. BAY pretreated mice were monitored daily for adequacy of food, water, bedding and general overall health conditions. No adverse effects were recorded.

Animals were maintained on 12-h light/dark cycles with free access to water. Clinical signs of sepsis including mild closure of the eyes, diarrhea, ruffled fur, and lethargy were monitored. Mice were sacrificed by cervical dislocation 24 h after surgery. Following rapid midline thoracotomy, the heart was quickly excised and then submerged in ice-cold bicarbonate-buffered Krebs–Henseleit solution.

### 4.2. Mitochondrial Respiration in Permeabilized Cardiac Fibers

Mitochondrial respiration was carried out in permeabilized cardiac fibers as previously described [22]. After quick dissection, the heart was placed into Biopsy preservation solution (BIOPS) relaxing solution containing 2.77 mM CaK_2_EGTA, 7.23 mM K_2_EGTA, 6.56 mM MgCl_2_, 0.5 mM dithiothreitol, 50 mM K-MES, 20 mM imidazole, 20 mM taurine, 5.3 mM Na_2_ATP, 15 mM phosphocreatine (pH 7.1 at 4 °C). Fiber bundles were dissected and transferred into a BIOPS solution containing 50 μg/mL saponin for 30 min. Permeabilized fibers were then rinsed three times with MiR05 mitochondrial respiration buffer containing 110 mM sucrose, 20 mM 4-(2-hydroxyethyl)-1-piperazineethanesulfonic acid (HEPES), 10 mM KH_2_PO_4_, 20 mM taurine, 3 mM MgCl_2_·6H_2_O, 60 mM MES-K, 0.5 mM ethylene glycol-bis(β-aminoethyl ether)-*N*,*N*,*N*′,*N*′-tetraacetic acid (EGTA) and 0.1% bovine serum albumin (pH 7.1 at 4 °C). Approximately 2 mg of wet weight cardiac fibers were placed in the chambers of oxygraph-O2k respirometer (Oroboros Instruments, Innsbruck, Austria). State 2 respiration Complex I-dependent “state 2” respiration (V_glut+mal_) was assessed by addition of 10 mM l-glutamate + 2 mM l-malate in absence of ADP. Complex I-dependent “state 3” respiration (V_glut+mal+ADP_) was achieved by adding 5 mM ADP. The respiratory control ratio (RCR) was calculated as state 3/state 2 ratio, which was used to estimate the capacity of mitochondria to oxidize respiratory substrates and to synthesize ATP. Absence of significant respiration increases following addition of cytochrome c (10 μM) confirmed intactness of the outer mitochondrial membrane. At the end of the experiments, respirometer sensors were calibrated for zero O_2_ content using dithionite. Respirometer chambers were thermostated at 24 °C and rates of respiration were calculated in pmol O_2_·s^−1^·mg·wt^−1^.

### 4.3. CO_2_-Generating α-Ketoglutarate Dehydrogenase Complex

Permeabilized cardiac fibers were exposed to the CO_2_-generating combination of α-ketoglutarate dehydrogenase complex (KGDHC), its substrates ketoglutaric acid + NAD^+^, and its cofactors coenzyme-A and cocarboxylase [23]. KGDHC activity was initiated in the 2 mL oxygraph chambers using MiR05 respiration medium (24 °C) containing 0.3 mM cocarboxilase (Thiamine pyrophosphate TPP, Sigma-Aldrich (Lyon, France), 0.5 mM β-Nicotidamide Adenine Dinucleotide (β-NAD+, Sigma-Aldrich), 0.24 mM coenzyme A (Sigma-Aldrich), 5 mM α-ketoglutarate (Sigma-Aldrich), and 1 mM dithiothreitol (DTT, Sigma-Aldrich). Next, 0.5 units of KGDHC (Sigma-Aldrich) were added to the 2 mL chambers. After 3 min of reaction, permeabilized cardiac fibers were then added to the medium. CcOX driven respiration was evaluated in 0.5 mM *N*,*N*,*N*′,*N*′-tetramethyl-*p*-phenylenediamine dihydrochloride (TMPD)/2 mM ascorbate dependent respiration measured after blocking complex III with antimycin A (1.5 μM).

### 4.4. cAMP Measurement

For cAMP measurement, tissues were collected 24 h after sham and CLP surgery. Heart pieces (*n* = 6 hearts for each group), equilibrated in aerated (95% O_2_, 5% CO_2_) Krebs–Henseleit bicarbonate buffer for 30 min at 37 °C, were exposed to PDE inhibitors for the next 30 min, and quickly snap frozen in liquid nitrogen. Both basal and PDE inhibitor–stimulated tissue cAMP levels were determined. Liquid nitrogen snap frozen heart tissues were ground into powder, homogenized in 10 volumes of 0.1 M HCl and centrifuged at 1500× *g* for 10 min. Supernatant, thus obtained, was neutralized used for cAMP assay using an ELISA kit following the manufacturer’s instructions (Enzo Life Science, Farmingdale, NY, USA). Concentrations of cAMP were expressed as pmol/mg protein.

### 4.5. Isolated Perfused Heart Preparation

Freshly excised mouse hearts were placed into ice-cold Krebs–Henseleit buffer solution and immediately mounted onto a Langendorff apparatus. Hearts were perfused in a retrograde fashion via the aorta at a constant flow rate of 2 mL/min with aerated (95% O_2_, 5% CO_2_) Krebs–Henseleit (KH) bicarbonate buffer containing NaCl 120 mM, KCl 4.8 mM, KH_2_PO_4_ 1.2 mM, MgSO_4_ 1.2 mM, NaHCO_3_ 25 mM, CaCl_2_ 1.25 mM, glucose 11 mM (37 °C; pH 7.35–7.40). Coronary flow was generated by a calibrated peristaltic pump (Masterflex, Fisher Scientific, Paris, France) and monitored by a transit-time ultrasound flow-meter (Transonic, Ithaca, NY, USA) with size-1 PXN Inline flow sensor. Cardiac contractile function was assessed using a metal hook inserted into the heart apex to control and record developed force and heart rate. Force was recorded continuously with a transducer (MT0201; ADInstruments Ltd., Paris, France), digitized online at a sampling frequency of 400 Hz and recorded for analysis (Chart version 7 Pro; ADInstruments Ltd., Paris, France). Transducers were calibrated and connected to an ML224 bridge amplifier that fed into a Powerlab 8SP data acquisition system (ADInstruments Ltd., Paris, France). Hearts were stretched to 1 gram-force (≈10 milli-Newtons—mN) and paced at 9 Hz with 5 ms square pulses at a voltage of 10 volts. The resting tension was then increased by 0.25 gram-force increments until optimal mechanical performance of the length-active isometric tension curve. After 30 min of equilibration, left ventricle developed force (LVDF), i.e., the difference between resting and peak force, was calculated and averaged over 50 consecutive contractions for analysis. Oxygen partial pressures were measured in the aerated Krebs–Henseleit buffer (PO_2_ perfusate; “inflow”) and the coronary effluent collected with a catheter placed in the pulmonary artery (PO_2_ effluent; “outflow”) using electrodes connected to a blood gas analyzer (ABL 720 Radiometer SAS, Neuilly Plaisance, France). Hearts were weighed at the end of the experiments. Myocardial O_2_ uptake (MVO_2_) in μL·min^−1^·g^−1^ was using standard formula with specific coefficients of O_2_ solubility and O_2_ density at 37 °C (MVO_2_ = coronary flow (μL·min^−1^)/gram of heart tissue × (PO_2_ perfusate − PO_2_ effluent) (mmHg) × oxygen solubility. (760^−1^) (24 μL/mL O_2_ at 37 °C). LVDF × heart rate divided by MVO_2_ was calculated as cardiac efficiency.

### 4.6. Western Blotting

Flash frozen hearts were homogenized at 4 °C in radio-immunoprecipitation assay (RIPA) buffer (in mmol·L^−1^: Tris 10, NaCl 140, EDTA 5, phenylmethanesulfonylfluoride PMSF 1 with Triton X-100 1%, deoxycholate 1%, sodium dodecyl sulfate (SDS) 0.1%, and in μg/mL: aprotinin 10, leupeptin 10, pepstatin 10, at pH 7.4) using a glass dounce tissue grinder. A mitochondria isolation kit for tissue (Abcam, Paris, France) was used to prepare enriched mitochondrial fractions, which were stored at −80 °C until use. Proteins (25 μg) were resolved by SDS-polyacrylamide gel electrophoresis (PAGE), transferred onto a nitrocellulose membrane and incubated with the following antibodies: rabbit polyclonal anti VDAC-1 antibody (1/1000; Abcam, Paris, France), rabbit polyclonal anti-phospho-Ser-58 CcOX IV-1 antibodies (1/500; Phospho Solutions, Aurora, CO, USA). Blots were developed with enhanced chemiluminescence (ECL) Plus reagent with goat anti-rabbit IgG antibody conjugated to horseradish peroxidase for chemiluminescent detection (Cell Signaling Technology, Beverly, MA, USA).

### 4.7. Titration of Agonists and Inhibitors

Agents modulating mitochondrial cAMP-PKA pathway used in our study are summarized in Figure 1. Drugs were titrated to determine conditions that resulted in mitochondrial respiration and cardiac performance optimal effects. For mitochondrial respiration studies with permeabilized cardiac fibers, agonists/inhibitors were used as follows: incubation for 30 min with 8Br-cAMP 3 mM (Sigma-Aldrich), H89 50 μM (Calbiochem, San Diego, CA, USA), forskolin 10 μM (Sigma-Aldrich), KH7 5 μM (Cayman Chemical Company, Ann Arbor, MI, USA), 3,7-dihydro-1-methyl-3-(2-methylpropyl)-1*H*-purine-2,6-dione IBMX 100 μM (Sigma-Aldrich) or 2-((3,4-dimethoxy phenyl)methyl)-7-((2*R*,3*R*)-2-hydroxy-6-phenylhexan-3-yl)-5-methyl-1*H*-imidazo (5,1-f)(1,2,4)triazin-4-one BAY 60-7550 100 nM (Cayman Chemical Company). Isolated perfused hearts were perfused with 10–100 μM of IBMX or with 10–100 nM of BAY 60-7550 for 30 min before measurements.

For cAMP measurement, heart pieces were either exposed to 100 μM IBMX and 10 μM forskolin or 100 nM BAY 60-7550 for 30 min, and then quickly snap frozen in liquid nitrogen. For Western blot experiments in enriched mitochondrial fractions, 8Br-cAMP was used at different incubation times (10 to 60 min) and doses (3 to 30 mM) to study Ser-58-phosphorylated CcOX subunit IV-1 protein levels. Thereafter, incubations of 3mM 8Br-cAMP, 50 μM H89 and 100 nM BAY 60-7550 for 30 min were used in Western blot studies. For in vivo experiments, either BAY 60-7550 or solvent were administered as pre-treatment (1 mg/kg intraperitoneal (i.p.) injection per day for 3 days prior to surgery) or treatment (0.5, 1 and 5 mg/kg i.p. immediately before surgery). Drug solvent dimethyl sulfoxide (DMSO) (0.1% in normal saline) was used as the vehicle in ex vivo and in vivo experiments.

### 4.8. Study Design

**Figure 8:**
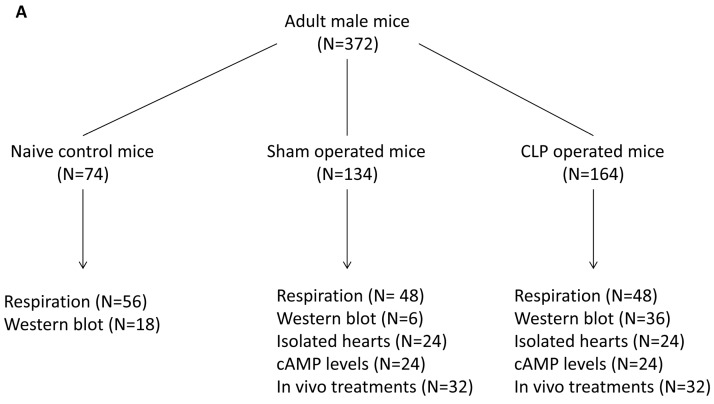

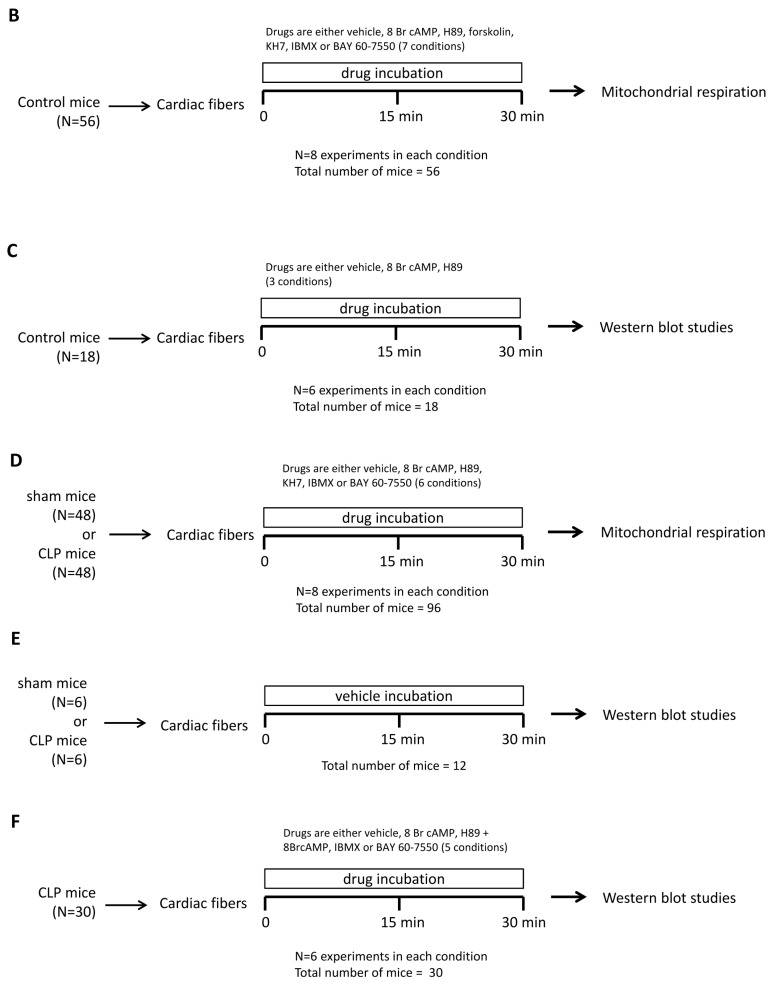

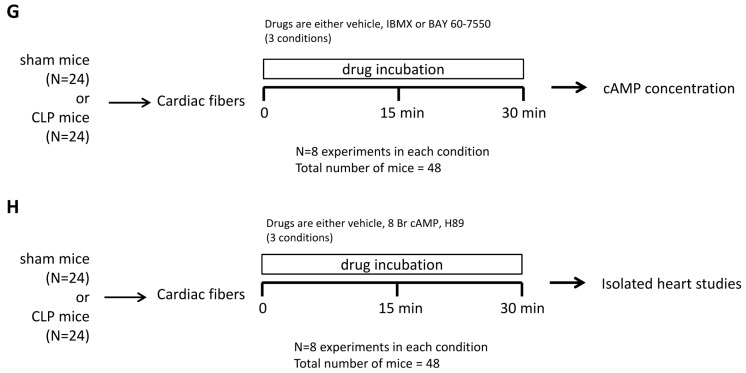
Total number of mice is displayed in (**A**). We first evaluated in vitro effects of vehicle, 8Br-cAMP, H89, KH7, IBMX; and BAY 60-7550 on mitochondrial respiration of permeabilized cardiac fibers prepared from control naïve mice (**B**); in separate series of control cardiac fiber preparations, in vitro effects of vehicle, 8Br-cAMP and H89 incubation on CcOX subunit IV-1 and VDAC expressions were documented (**C**); second, we evaluated in vitro effects of vehicle, IBMX, BAY 60-7550, 8Br-cAMP, H89, and KH7 on mitochondrial respiration of permeabilized cardiac fibers prepared from sham-operated and CLP-operated mice (**D**); in separate sets of cardiac fiber experiments, we evaluated the effects of CLP sepsis on CcOX subunit IV-1 and VDAC expressions (**E**); in addition, we evaluated the effects of in vitro incubation of 8Br-cAMP, IBMX and BAY 60-7550 on CcOX subunit IV-1 in septic cardiac fibers (**F**); third, we evaluated the effects of IBMX and BAY 60-7550 on cAMP cardiac levels in sham-operated and CLP-operated mice in a separate set of experiments (**G**); eventually, effects of IBMX and BAY 60-7550 in ex vivo hearts isolated from sham-operated and CLP-operated mice were tested (**H**).

Study design is summarized in Figure 8.

### 4.9. Statistical Analysis

For mitochondrial respiration studies, sample size calculation was determined a priori by using previous results from our laboratory. We anticipated that eight samples per experiment would have been needed to detect 20% difference between groups with α = 0.05 and β = 0.10, yet this calculation was based on only 2 groups. Results were analyzed with the SPSS for Windows software (SPSS 15.0, IBM Analytics, Paris, France). Results were expressed as mean ± SD. Normality of distribution was verified a posteriori by using the Shapiro–Wilk test, which allowed us to run multiple comparisons with post hoc Bonferroni tests with 6 numbers of comparisons per family. When a significant difference was found, we identified specific differences between groups using Bonferroni post hoc analyses. Ajusted *p*-value was considered for statistical significance.

## 5. Conclusions

In conclusion, the results of our study suggest that mitochondrial cAMP-PKA signaling is impaired in septic cardiac fibers, as evidenced by mitochondrial respiration and PKA-dependent cytochrome c oxidase phosphorylation decreases. PDE2 inhibition increased respiratory control ratio in septic cardiac fibers and improved cardiac MVO_2_ efficiency in septic heart preparations. Pretreatment with PDE2A inhibitor in vivo partially improved sepsis-induced myocardial dysfunction in mice.

## Figures and Tables

**Table 1:** Effects of phosphodiesterase inhibitors on mitochondrial respiration in permeabilized cardiac fiber preparations.

Respiration Parameters	Sham	Sepsis
V_glut+mal_ respiration rate		
vehicle	14.5 ± 1.7	16.3 ± 2.3
IBMX	15.8 ± 4.2	14.9 ± 4.2
BAY 60 7550	15.1 ± 3.7	10.3 ± 5.1 ^#^
V_glut+mal+ADP_ respiration rate		
vehicle	103.6 ± 14.7	87.7 ± 4.5 *
IBMX	101.9 ± 28.0	79.1 ± 30.3 *
BAY 60 7550	93.4 ± 20.9	84.4 ± 29.1 *
Respiratory control ratio		
vehicle	4.67 ± 0.91	3.18 ± 0.40 *
IBMX	4.46 ± 0.59	3.11 ± 0.28 *
BAY 60 7550	4.35 ± 0.82	3.88 ± 0.31 ^#^

Effects of ex vivo incubation of phosphodiesterase (PDE) inhibitors, non-mitochondrial membrane permeant 3,7-dihydro-1-methyl-3-(2-methylpropyl)-1*H*-purine-2,6-dione (IBMX) and mitochondrial-targeted BAY 60-7550 on mitochondrial complex I-dependent “state 2” (V_glut+mal_) respiration rates, complex I-dependent “state 3” (V_glut+mal+ADP_) respiration rates and respiratory control ratio, i.e., V_glut+mal+ADP_/V_glut+mal_ in permeabilized cardiac fibers of sham-operated mice and cecal ligation and puncture (CLP)-operated mice. All mice were sacrificed 24 h after surgery. See Materials and Methods for details. Rates of respiration are pmol O_2_·s^−1^·mg. Data are expressed as percent of change and displayed as mean ± SD. Results were analyzed with one-way ANOVA and Bonferroni’s multiple comparison post hoc adjustment (*n* = 8 in each group; * indicates *p* < 0.05 versus sham; ^#^ indicates adjusted *p* < 0.01 versus vehicle).

**Table 2:** Effects of phosphodiesterase (PDE) inhibitors on cardiac contractile function in isolated heart preparations.

Cardiac Parameters	Sham	Sepsis
**LVDF (gram-force)**		
(1) Ex vivo perfusion		
● vehicle	1.41 ± 0.28	0.53 ± 0.34 *
● IBMX	2.24 ± 0.37 ^#^	1.28 ± 0.20 *^,^^#^
● BAY 60 7550	1.53 ± 0.82	1.05 ± 0.25 *^,^^#^
(2) In vivo BAY 60 7550	1.51 ± 0.28	0.62 ± 0.31 *
**Coronary pressure (mmHg)**		
(1) Ex vivo perfusion		
● vehicle	88 ± 14	86 ± 17
● IBMX	85 ± 3	88 ± 6
● BAY 60 7550	84 ± 23	84 ± 25
(2) In vivo BAY 60 7550	85 ± 14	52 ± 23 *^,^^#^
**MVO_2_ (μL·min^−1^·g^−1^)**		
(1) Ex vivo perfusion		
● vehicle	185 ± 14	95 ± 23 *
● IBMX	275 ± 42 ^#^	205 ± 25 *^,^^#^
● BAY 60 7550	192 ± 23	142 ± 23 *^,^^#^
(2) In vivo BAY 60 7550	189 ± 14	97 ± 14 *
**Efficiency**		
(1) Ex vivo perfusion		
● vehicle	4.1 ± 0.85	3.0 ± 0.85 *
● IBMX	4.3 ± 0.57	3.3 ± 0.57 *
● BAY 60 7550	4.3 ± 0.28	4.0 ± 0.57 ^#^
(2) In vivo BAY 60 7550	4.4 ± 0.85	3.8 ± 0.85 ^#^

Effects of ex vivo perfusion (IBMX, BAY 60 7550) and in vivo treatment (BAY 60 7550) on cardiac contractile function evaluated in isolated heart preparations of sham-operated mice and cecal ligation and puncture CLP-operated mice. Left ventricular developed force LVDF: difference between peak and resting force (1 gram-force ≈ 10 millinewtons; mN); MVO_2_: myocardial O_2_ uptake; Efficiency: LVDF × heart rate divided by MVO_2_. Ex vivo perfusion: data are presented under basal conditions (vehicle) and after PDE inhibition with either IBMX (100 μM) or BAY 60-7550 (100 nM). In vivo BAY 60 7550: pretreatment of mice with BAY 60 7550. See Materials and Methods for details. Data are mean ± SD. Results were analyzed with two-way ANOVA and Bonferroni’s multiple comparison post hoc adjustment (*n* = 8 per group; * indicates *p* < 0.05 versus sham; ^#^ indicates adjusted *p* < 0.01 versus vehicle).
